# Correlative tomography and authentication features of a shrunken head (*tsantsa*)

**DOI:** 10.1371/journal.pone.0270305

**Published:** 2022-08-03

**Authors:** Lauren September Poeta, Maria Patricia Ordóñez, Eric Fournier, Andrew John Nelson

**Affiliations:** 1 Department of Anthropology, Western University, London, Ontario, Canada; 2 Colegio de Ciencias Sociales y Humanidades, Universidad San Fransisco de Quito, Quito, Ecuador; 3 ORS, Montreal, Quebec, Canada; Liverpool John Moores University, UNITED KINGDOM

## Abstract

Museum personnel and the general public have become quite familiar with the presence of shrunken heads in museum collections, but the procedures to authenticate the history and origin of these unique cultural items are not yet reliable. These shrunken heads, called *tsantsas*, are meant to be the cultural material remains of ceremonies conducted by the Shuar and Achuar Peoples of South America. This project seeks to integrate the use of micro-computed tomography (micro-CT) scanning with methods used in previous studies (clinical computed tomography (CT) and visual inspections) to examine authentication procedures of shrunken heads (*tsantsas*) held in contemporary museum collections. We use a correlative tomographic approach using several scans at successively higher resolutions to determine whether a *tsantsa* was created from human remains, and if so, what key features can best contribute to its authentication. Conclusively, our correlative tomographic approaches provide new insights into the determination process of whether a *tsantsa* was created from real human remains or not. Also, this study questions whether the previously conceptualized dichotomy of ceremonial or commercial might be better thought of as a continuum of practice. Investigating and redefining the examination and authentication procedures of *tsantsas* is crucial for future ethical curation, management, and repatriation efforts of this unique cultural material of the Shuar and Achuar Peoples.

## Introduction

South American shrunken heads, some known as *tsantsas*, are common in many museum collections. However, it is currently difficult to identify whether they are authentic, including whether they were created from human remains. *Tsantsas* were either created for ceremonial purposes (considered to be “authentic”) or for the commercial market (not considered to be “authentic”). The anthropological literature has reported ceremonial tsantsas as war trophies of enemy heads that were collected after battle and shrunken by the Shuar and Achuar People of Ecuador and Northern Peru in an attempt to trap the avenging soul of the deceased and protect the victor from spiritual revenge [1–13]. Conflicting discourses exist in the current literature about whether the *tsantsas* should be attributed solely to the Shuar [6], the entire Chicham linguistic group (SAAWC) [14], or only the Shuar and Achuar of the Chicham group [4]. However, recent studies and active community engagement regarding these remains have challenged the remains’ uses and origins [4–6, 9]. A belief exists among contemporary Shuar communities that *tsantsas* may have also been made from Shuar clan leaders who died of natural causes, this would have harnessed their power positively for the community going forward and shown respect to past leaders [6, 15, 16]. This Indigenous knowledge goes against what has been previously published in *tsantsa* literature, that *tsantsas* are only created from enemies killed in warfare [6]. Early literature reports that *tsantsas* are believed to contain *tsarutama*, a vitalized, magical power [7]. The *arutam*, or powerful soul of the deceased, is thought to see and speak for both good and evil [11].

By creating a tsantsa, the soul is trapped inside the remains as the eyes and mouth are closed so there can be no unintended impact on the living from the spirit’s power [17]. *Tsantsas* were then displayed inside specific houses or on poles [18], and not worn as suggested in much of the existing anthropological literature (e.g. Castner, 2002). Ethnohistoric evidence suggests that *tsantsas* were being created as early as the 1500s [7, 14]. In the early- to mid-1800s, the Shuar and Achuar People developed trading relationships with nearby European settlers and missionaries, especially Salesians, who entered the area for “appeasement” or Christianisation missions which included separating children from their parents [4, 12]. Given the Salesian’s forceful and colonial acculturation processes, many of the traditional practices of the Shuar and Achuar were forgotten or changed by the 1940s, including practices of *tsantsa* production [4]. This trade in cultural artifacts included shrunken heads, which the priests, Victorian-era Europeans, and American explorers acquired as curios and collectables for their private, worldly collections. After 1860, scientific communities of North America and Europe began publishing on the practice of head shrinking [1, 2], leading to worldwide recognition of this practice.

The commercial production of *tsantsas* and their subsequent worldwide distribution soon began in order to satisfy a growing demand for *tsantsas* as curios and as ethnographic artifacts for museums [4, 8, 10–12, 19]. Commercial *tsantsas* were often made from animal skins, including pigs, monkeys, and sloths [10, 19, 22]. These animal skins could mimic human morphological features by being softened in hot water and then moulded over a wooden form [19]. As commercial collectors desired shrunken human heads for display and not ritual purposes, commercial *tsantsas* required lifelike human features more than their ceremonial counterparts [14, 18, 20–22]. Consequently, real human heads are said to have been documented in commercial production to increase profits from their “realistic features” and were reportedly sourced from unclaimed bodies in local hospitals [7, 19, 21], but this remains a point of contention as the only source of available evidence for this practice is sensationalized news articles [4]. However, ethnohistoric accounts also cite animal skins, particularly sloths, being used in ceremonial contexts [3, 7, 10, 11, 19, 22], occasionally using plugs of human hair as a wig [10]. This makes investigative efforts difficult, as both origin and material become very complex questions.

Unfortunately, there are no accounts written by the Shuar and Achuar Peoples themselves that detail the ritual process, but contemporary work is beginning to acknowledge the existing oral histories and Indigenous knowledge previously overlooked [4, 6]. While European and American written ethnohistorical documents exist [e.g.1-3, 7, 23, 24], they include conflicting accounts of both ceremonial and commercial preparation methods. Since commercial *tsantsa* manufacturers claimed their *tsantsas* were real, contemporary collections worldwide contain a range of ceremonial and commercial *tsantsas*, up to 80% of commercial origin [14], with few reliable methods to differentiate their true origin.

Although ethnohistorical accounts often conflict, a basic consensus narrative can be constructed based on overlapping details in the published accounts of the preparation of ceremonial *tsantsas*. When enemies’ heads were recovered, they were removed from the body at the base of the neck [14, 17, 19]. The heads were then strung on a vine through the mouth and esophagus, or placed in a reed basket, to be carried to a production site away from enemy territory [10, 14, 17, 19]. Some literature suggests that the removal of the skull was always done by a median posterior incision from the crown down the neck, with the skin then peeled off the skull [14, 17, 19]. However, other ethnohistorical accounts note variations, including the location of a single incision, multiple incisions [7], or the breaking of the bones of the skull to aid in the removal of smaller bone fragments [24, 25]. However, recent workshops with Shuar and Achuar leaders at the Universidad San Francisco de Quito concluded that ceremonial *tsantsas* were only created using a single incision [4, 5, 9, 26]. All incisions were crudely done with sharpened bamboo, shells, or a lithic blade in ceremonial contexts, while commercial producers may have used finer tools, such as sharp steel knives [3, 7, 11, 20, 24, 25]. However, ethnohistorical accounts also indicate that neat incisions created using sharp pieces of bamboo were possible [27]. The boney skull was discarded into the river beside the preparation site [7, 13]. The skins were then boiled for fifteen minutes to two hours, extracting fat and grease from the head as it floated to the top of the water [8, 10, 21, 26]. Boiling caused the skin to contract and thicken while killing the microbes that cause decomposition [1]. The inside of the skin was scraped to remove connective tissues while it was still pliable [7, 18].

The eyes were sewn shut while the skin was turned inside out [14, 18, 27]. The posterior incisions and facial features were sewn shut with *chambira* (*Astrocaryum chambira* palm) fibres and a sharp wooden needle [6, 10, 14, 18]. Some accounts indicate the lips were shut initially with chonta (*Juania australis* palm) pegs which were then replaced with chambira fibres securing them in place for the shrinking process [16, 23–25, 27]. The replacement could also happen later at the great feast [1]. However, many ceremonial *tsantsas* in collections still have bamboo shoots in the lips, so chambira fibres may have been chosen by the individual *tsantsa* producers. Ears and noses were the only remaining facial openings, but these could be blocked with any available substance [10], but blocking was uncommon for ceremonial practices. However, ear adornments are common, featuring wooden tubes, feathers, beetle wings, or bamboo lobe expanders that were used to accentuate the lobes stretched by a person in their life [10, 28]. The rim of the base of the neck was sewn with *chambira* vine fibre to reinforce the opening and act as a handle while the head was too hot to hold in the next stage [10, 14, 18, 27]. Ordóñez has determined that commercial *tsantsas* were commonly held with pincer-like instruments when moulded due to the heat, creating another unique commercial production signature (Ordóñez personal observation 2021).

Small rocks were heated over a fire and rolled around inside the head to distribute the heat [3, 7, 8]. After using the heated stones, hot sand would be introduced to heat the smallest spaces inside the head [1, 7, 10]. The head was only filled halfway and was kept in constant motion during this production stage, so the heat was evenly distributed [1, 27]. The hot sand would shrink the head and draw out the remaining moisture to desiccate the head [21]. Through inspection of several *tsantsas*, Ordóñez determined that the sand and stones can sometimes still be found inside the head. In a recent repatriation case, researchers at Mercer University found newspaper from the 1940s filling the interior cavity [29]. In ceremonial *tsantsas*, the external anatomy of the ear and the auditory canal was preserved during the manufacturing process, including stretched ear lobes [18, 20, 23, 28, 30]. Once the maker was satisfied with the size, typically a quarter of the original size or the size of a fist [20, 25, 27], flat stones were heated to iron the skin’s outer surface while simultaneously burning the smaller facial hairs away [10, 14, 27]. However, this step did not always occur as some early ceremonial *tsantsas* still have facial hair (Ordóñez personal observation 2021).

Ceremonial *tsantsas* are also likely to be prognathic in appearance, with pronounced chins and flared nostrils [7, 23]. Most tsantsas’ slightly laterally compressed face and dolichocephalic head are attributed to the face being held in the maker’s palm while the thumb and fingers compressed the temples [8, 21]. Charcoal was often rubbed into the skin, darkening it, so the avenging spirit could not see out [1, 10, 11, 21]. There are various accounts of how and when charcoal was introduced, which could explain skin colour variation that is observed among different *tsantsas* [10]. Since *tsantsas* were meant to hang, two small holes were then created through the crown of the head to thread braided *chambira* fibres [10, 16]. While previous literature states that the face of a ceremonial *tsantsa* may be painted to resemble the man’s face [10, 16], Ordóñez’s workshops with Shuar and Achuar leaders have confirmed this is a sign of commercial production.

Features identified in this consensus account have been used to propose nineteen features thought to differentiate ceremonial from commercial *tsantsas* [e.g. 14, 18], including observations about tissue conservation, production techniques, and specific anatomical features (S1 Table). Previously, visual examinations and clinical computed tomography (CT) have been the key methods used for examination and authenticity assessments. However, four of the nineteen features are poorly resolved by clinical CT for accurate assessment. Therefore, we decided to combine the use of clinical CT and high-resolution micro-CT scans at three different resolutions to assess the visualization of these features. This process is called correlative tomography [31].

## Materials and methods

**Fig 1:**
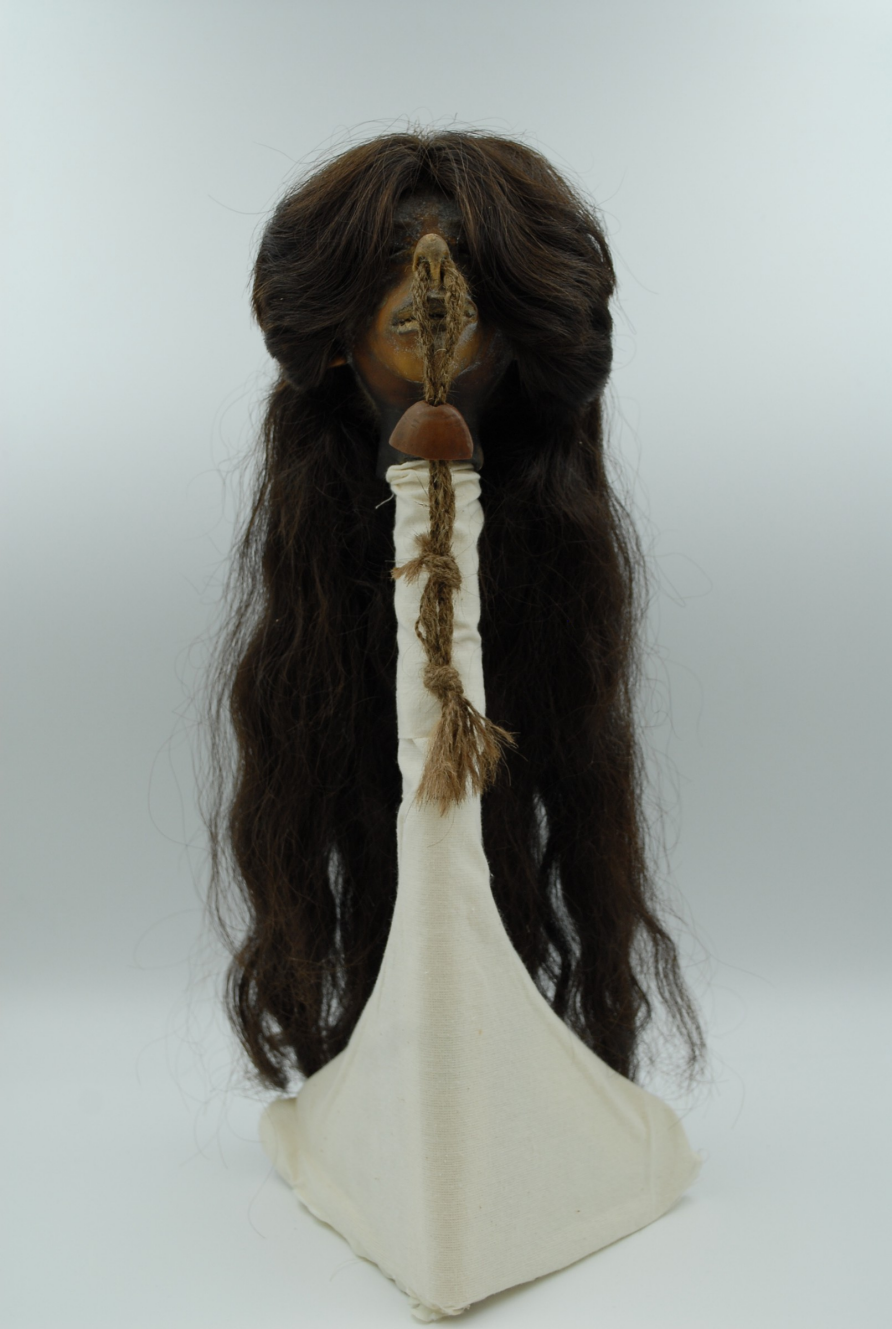
The Chatham *tsantsa*.

The *tsantsa* presented in this paper is in the collection of the Chatham-Kent Museum in Chatham, Ontario, Canada, and is referred to as the Chatham *tsantsa* throughout this paper (Fig 1). The Chatham *tsantsa*, Accession Number 1943.5.32, was introduced to the museum’s collections in the 1940s after the local Sulman family purchased the *tsantsa* while exploring the Amazonian Basin. The Sulman family did several world tours, and they also donated an Egyptian mummy and associated objects to the Chatham-Kent Museum [32]. The original accession record indicates that it came from “Peruvian Indians” but contains no additional information. A ‘Loans Outgoing Agreement’ was signed for the Chatham-Kent Museum for intended purpose of micro-CT scanning at the Museum of Ontario Archaeology. This loan was then verbally extended to include clinical CT scanning of the *tsantsa*. All necessary permits were obtained for the described study, which complied with all relevant regulations. Since the museum has no additional contextual information, the Chatham *tsantsa* exemplifies what can be discovered with similar context-lacking *tsantsas* in collections worldwide.

The *tsantsa* has a maximum cephalic length of 86mm and a maximum cephalic breadth of 66mm, yielding a cephalic index of 76.6, which is classified as dolichocephalic. The maximum length of the hair is 42cm and its length at the bangs is 12cm.

Computed tomography is the computerized compilation of x-ray projections that are reconstructed into a 3D volume that can then be “sliced” into 2D slices in any plane. 3D images can also be produced for viewing from any angle. In clinical CT scanners, slice thickness is the limiting factor for resolution, and minimum slice thickness typically falls within the range of 500–650μm [33]. However, in micro-CT scanners, the pixel size of the imaging panel is the limiting factor in resolution, and voxel (3D pixels) sizes of 1 to 50μm on a side can be achieved (depending on the size of the sample) [34].

This study required three separate computed tomographic scans. One clinical CT scan of the whole *tsantsa* was completed on the Canon Aquillon ONE at St. Joseph’s Healthcare. The other two scans were completed on a Nikon XT H225 ST micro-CT scanner at the Museum of Ontario Archaeology. One scan was of the whole head and the other was a high-resolution scan of a part of the tsantsa’s scalp. Both CT scanning facilities are in London, Ontario, Canada. Technical details of the three scans are presented in Table 1. The resolution of the whole head and scalp-only micro-CT scans is approximately 6.5 times and 35.7 times, respectively, that of the clinical scan. Both micro-CT scans were 53 minutes in length and captured 3141 projections through a 360^o^ rotation. 3D reconstructions based on the projections were completed using X-Tec CT Pro 4.4 software. All scan data was visualized, and the micro-CT data sets were registered and stitched using Dragonfly 4.1 and 2021.1 visualization software.

**Table 1:** Comparable image settings for the three scans.

	Clinical CT scan	Whole head micro-CT scan	Scalp only micro-CT scan
**beam energy**	80 kVp	87 kVp	87 kVp
**beam current**	800 mAs	95 μA	135 μA
**resolution**	500 μm slice thickness– 293 μm pixel size (X,Y planes)—anisotropic voxels	75 μm isotropic voxel size	14 μm isotropic voxel size
**data file(s) size**	971 MB	24.3 GB	21.5GB

## Results

When comparing the visualizations of the clinical and micro-CT scans, it is clear that even the highest resolution clinical CT scans are not able to allow adequate assessment of a *tsantsa’s* authenticity based on key manufacturing and anatomical characteristics. The assessment of stitching, eye and ear anatomy, and the scalp of the Chatham *tsantsa* are examined below to demonstrate the importance of CT resolution for trait visualization and why using multiple resolutions is crucial for complete assessments of *tsantsas*.

### Comparison of the stitching

**Fig 2:**
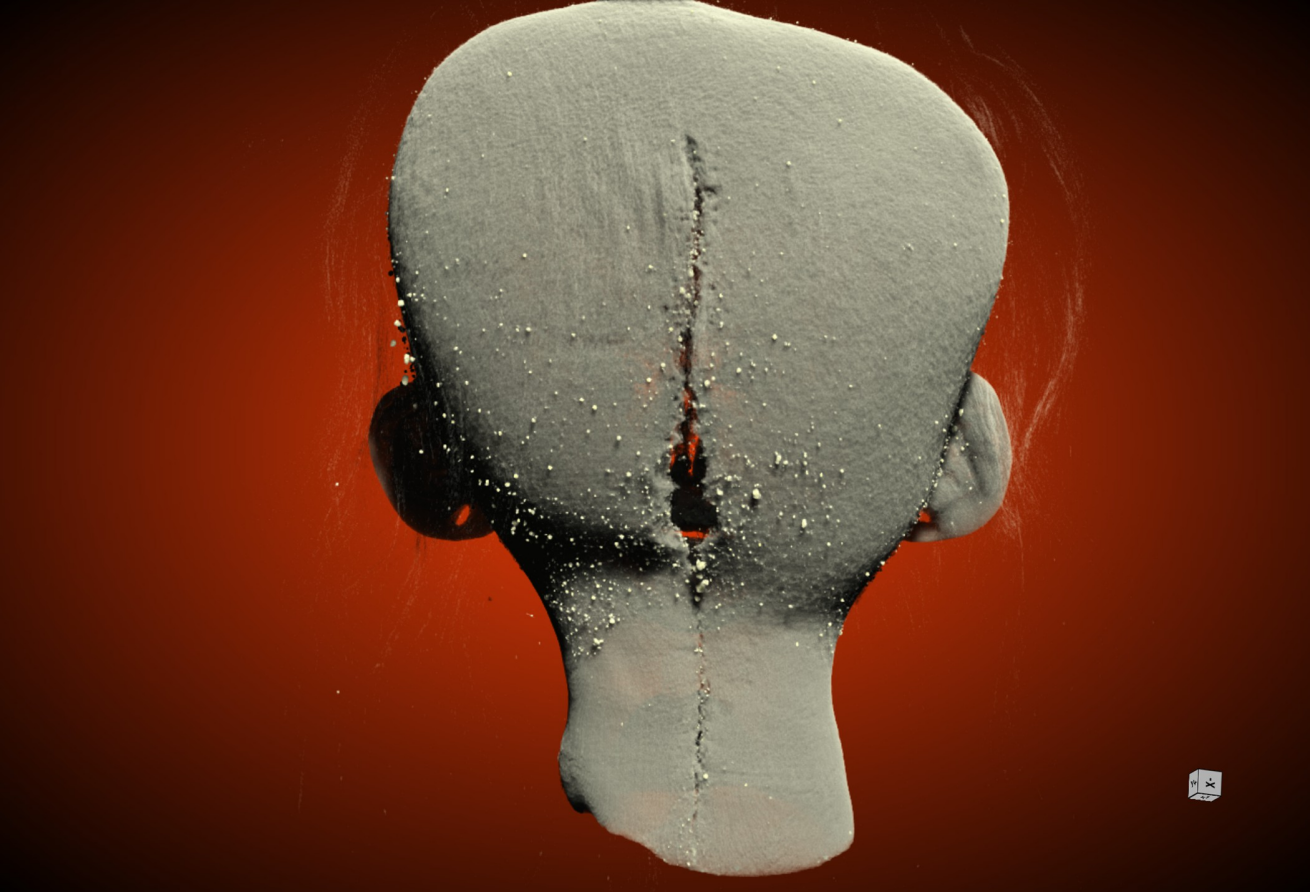
Digital visualization of the posterior incision and stitching. Micro-CT image of the incision at the rear of the skull, windowed and leveled to remove the hair.

**Fig 3:**
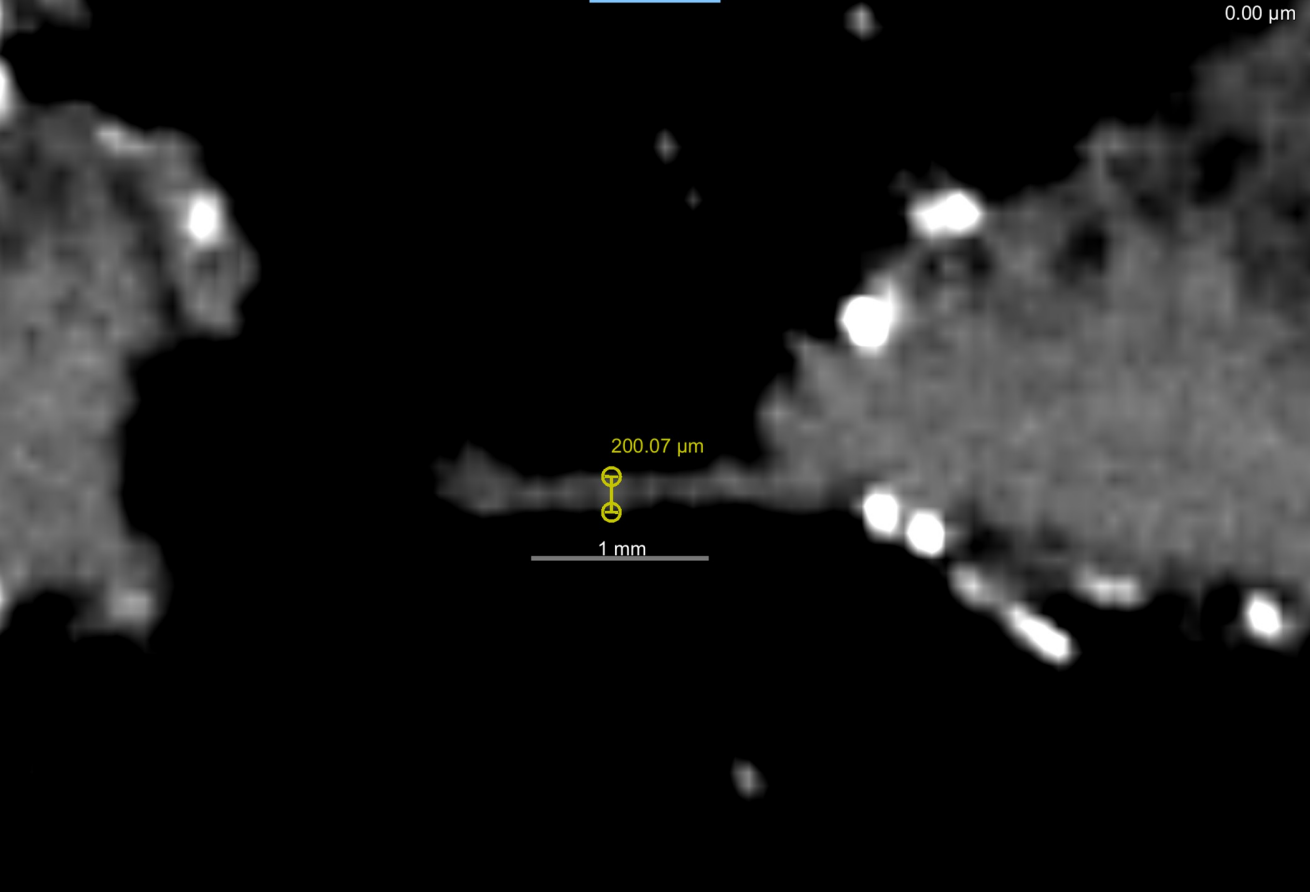
Digital visiualization of the posterior incision and stitching. Micro-CT image of a thread reaching across the posterior incision. Note the width (200 μm) and the fraying at the left end indicating that this is a thread and not chambira fiber.

There are differences when comparing the clinical CT resolution visualization to the micro-CT resolution visualization regarding the incision (Fig 2) and stitching of the neck and eye (Fig 3). The clinical resolution does not present clear margins of the incision itself, nor can it identify any stitching. With blurred margins of the incision, it is difficult to differentiate and assess intentional incision marks or damage from post-production handling. However, the micro-CT visualization (note: when micro-CT visualization of traits other than the scalp are being described, reference is being made to the 75 μm scan) offers a much more detailed depiction of the incision and enables the identification of stitching, that is not visible at clinical CT resolution. If there thread or fibres are present, exact measurements can be used to compare and possibly identify the material. In the case of the Chatham *tsantsa*, there is thread stitching the incision together and a single stitch at the rear part of each eye which have a diameter of 210μm. The thread corresponds to a multi-stranded heavy modern thread (V207, 200 μm [35]) as well as to the average thickness of an *A*. *chambira* fibre (204.8±17.5μm [36]). A close examination of a fragment of the thread shows that it is not smooth, as are chambira fibers [36], and it appears to be made of twisted threads that flair out at the broken end of a fragment at the posterior incision. The twisting is also present in the eye stitch (see below). Thus, this is likely to be modern thread rather than chambira fiber. A visual inspection of the inside of the neck incision demonstrates that dark-coloured glue has been used to keep the two halves of the incision together as well. While this is not a ceremonial practice, it could have been applied for conservation or museographic purposes any time post-production.

### Comparison of the eyes

**Fig 4:**
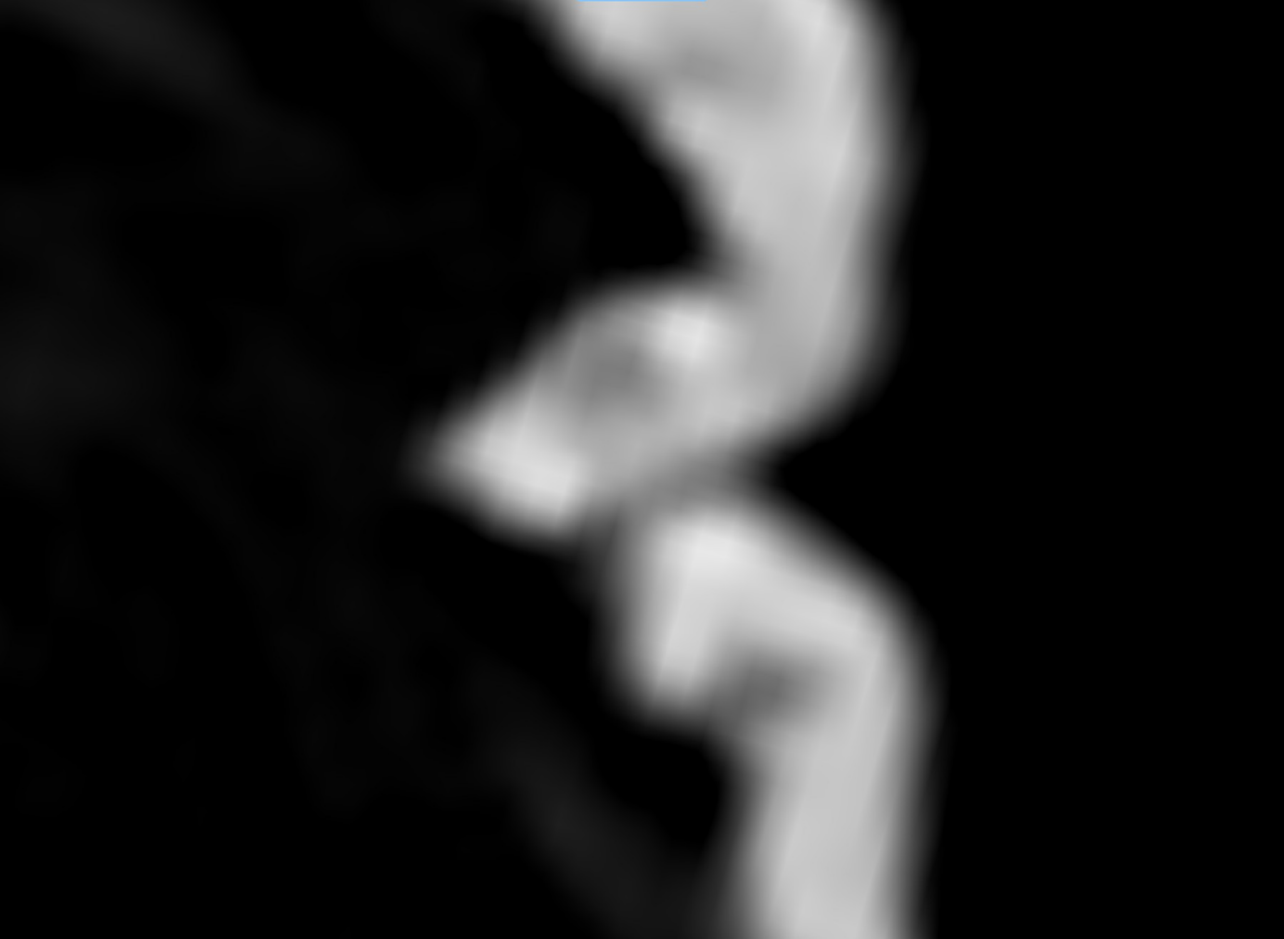
Comparative slice images of the eye at different resolutions. Clinical CT coronal slice through the eye.

**Fig 5:**
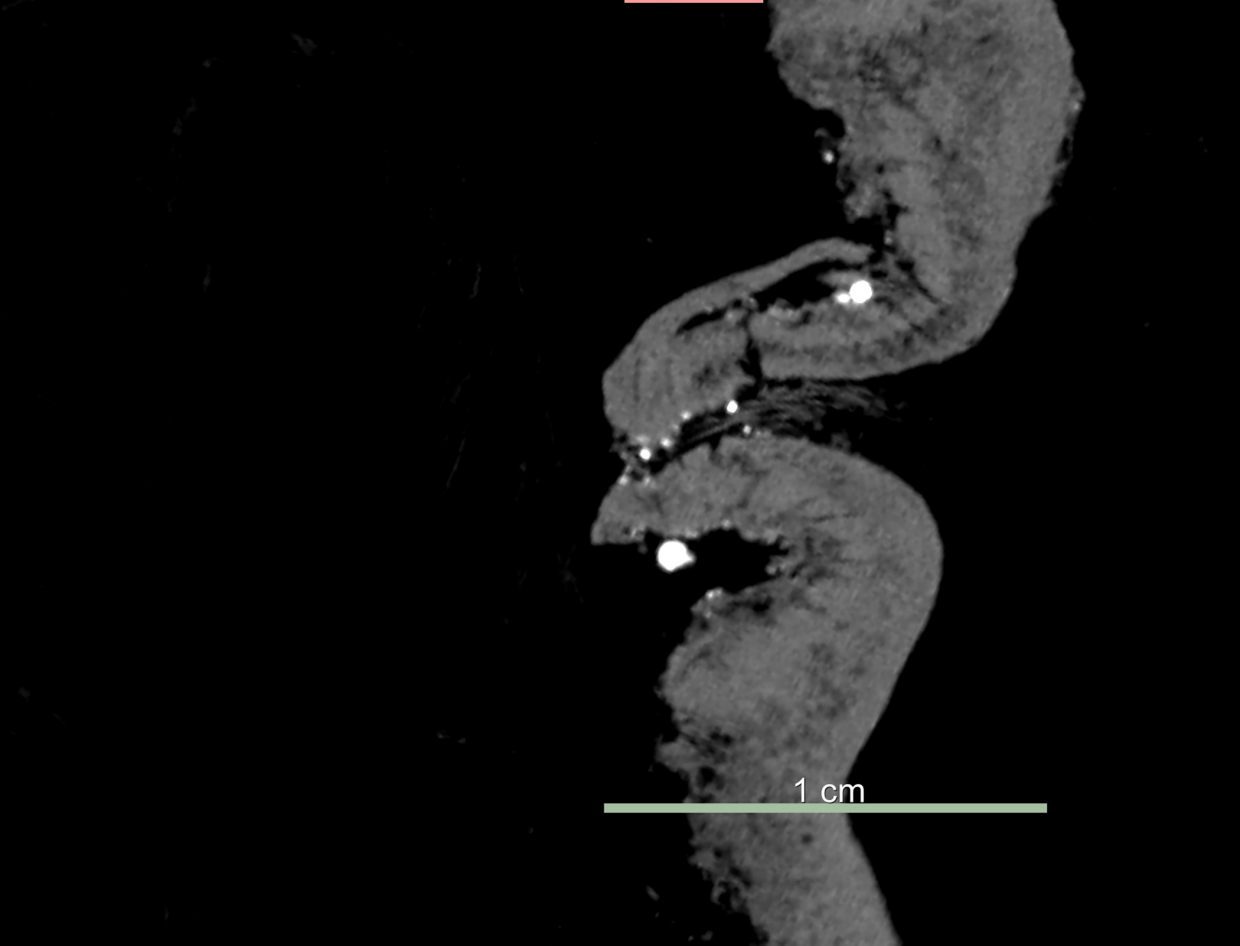
Comparative slice images of the eye at different resolutions. Micro-CT (75μm scan) coronal slice through the eye.

The eyelids of ceremonial *tsantsas* should be closed tightly and collapsing inwards, altering the natural shape of the eyes, which is, in life, determined by the orbital bones and eyeball. Sagittal sections of the Chatham *tsantsa* eyelids demonstrate both lids pointing inwards in the clinical CT and micro-CT whole head scans (Figs 4 and 5). However, the clinical CT resolution does not enable any further conclusive interpretations. Any identifying anatomical features, such as eyelashes (which can be seen as pointing outwards on visual inspection and on the micro-CT images) and tissue structure, are not visible at clinical resolution. The resolution is especially inadequate for assessing anatomical structure in the eye region as the low resolution blends the eyelids into one continuous piece of tissue. However, the micro-CT resolution resolves the individual tissue structures and anatomical detail not visible at clinical resolution. As mentioned above, there appears to be a single stitch with modern thread at the back of the eye which joins the upper and lower eye lids.

### Comparison of the ears

**Fig 6:**
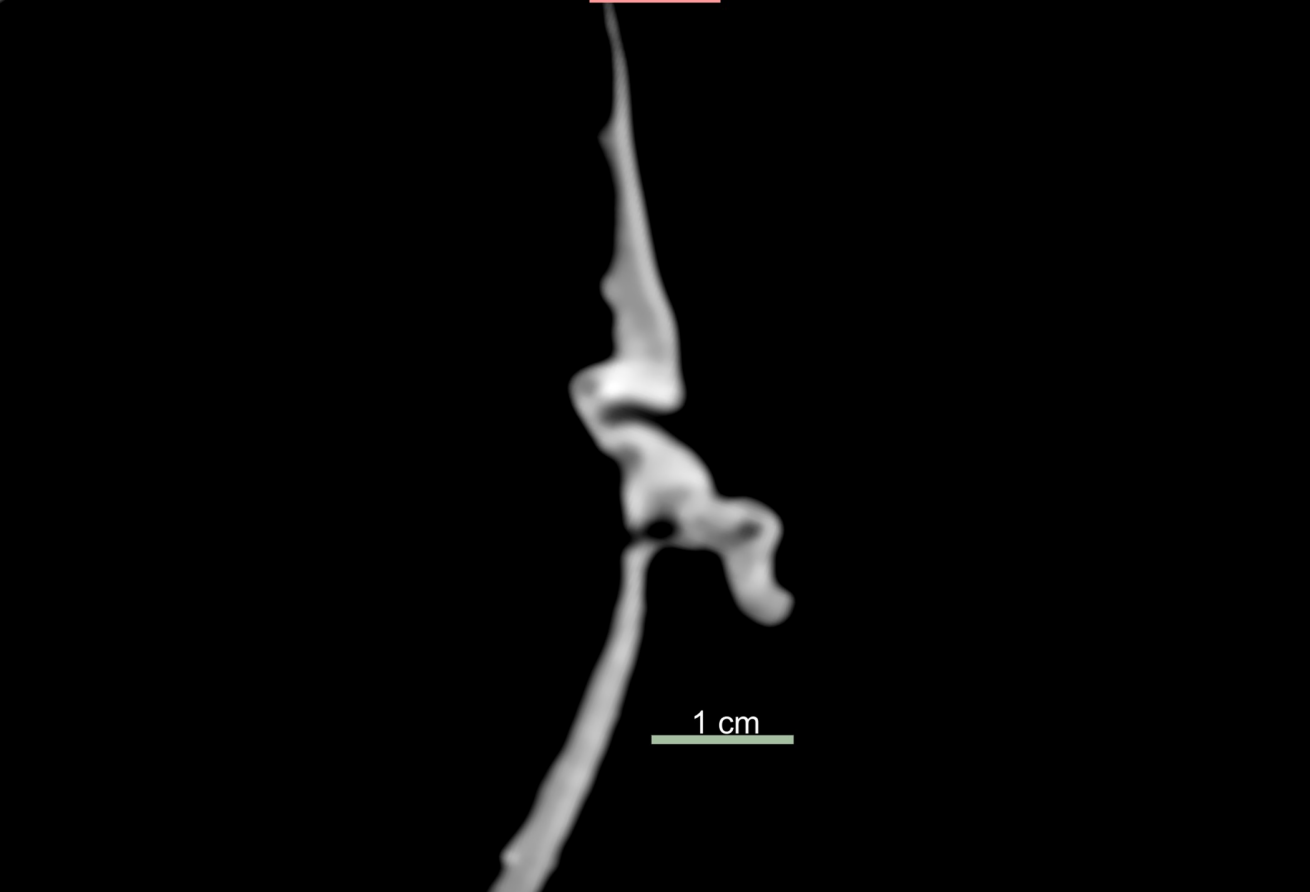
Comparative slice images of the right ear at different resolutions. Clinical CT axial slice of the right auditory canal and pinna.

**Fig 7:**
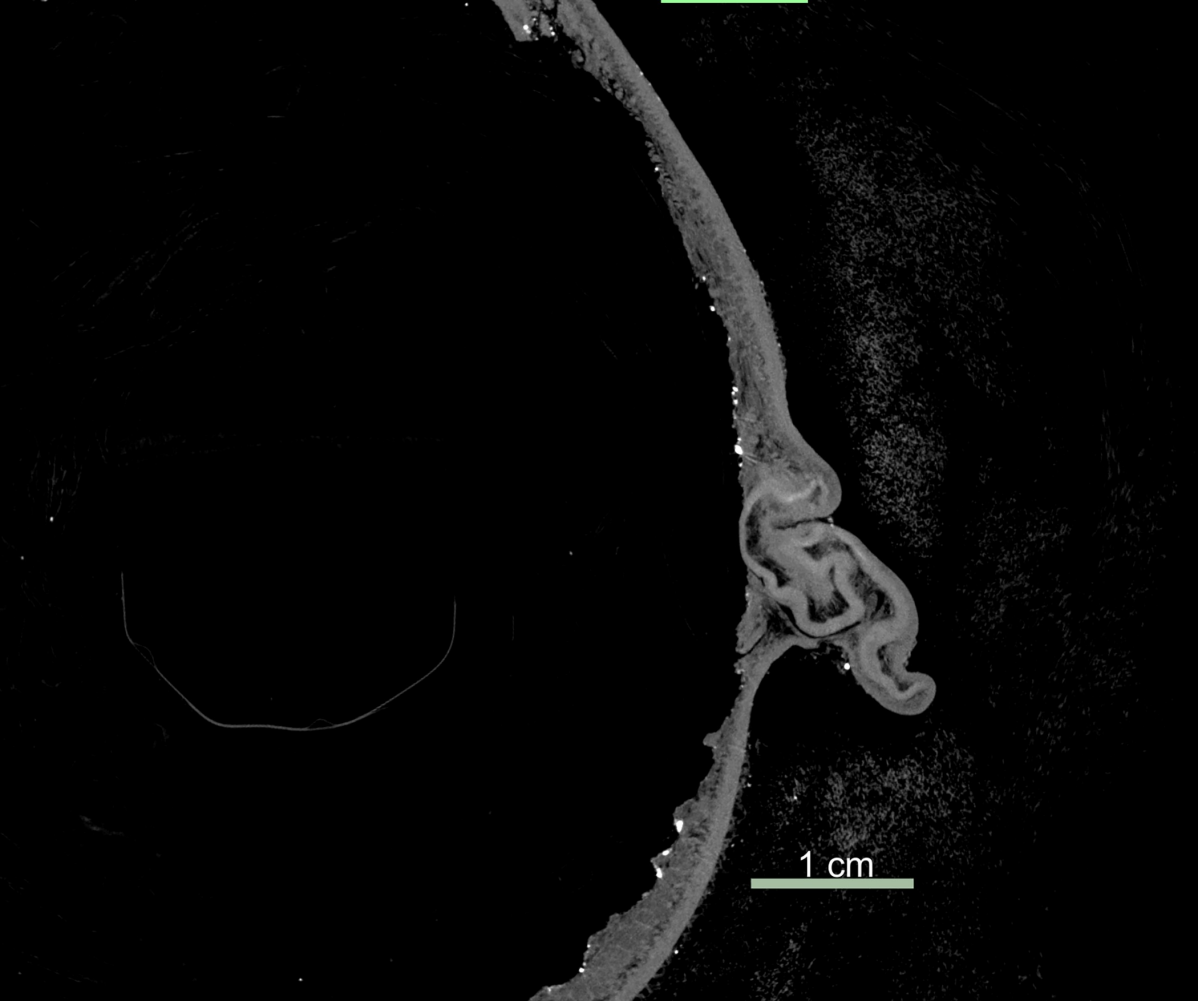
Micro-CT (75 μm scan) axial slice of the right auditory canal and pinna.

A comparison of the clinical CT and micro-CT scans demonstrates that there are benefits to the use of micro-CT in the visualization of the ear (Figs 6 and 7). Not only are the edges blurred like the eyes on the clinical scan, but it is difficult to assess the tissue structure to know if it is a true human ear or other tissue shaped to appear as an ear externally (Fig 6). Micro-CT imaging allows a detailed view of the anatomical structure of a *tsantsa’s* ear, including the external auditory canal, folds of cartilage and what appears to be the tympanic membrane (Fig 7). In addition, the micro-CT visualization demonstrates some dense inclusions, possibly quartz sand crystals deposited in the auditory canal and blocked by the tympanic membrane (Fig 4B).

### Comparison of the scalp

**Fig 8:**
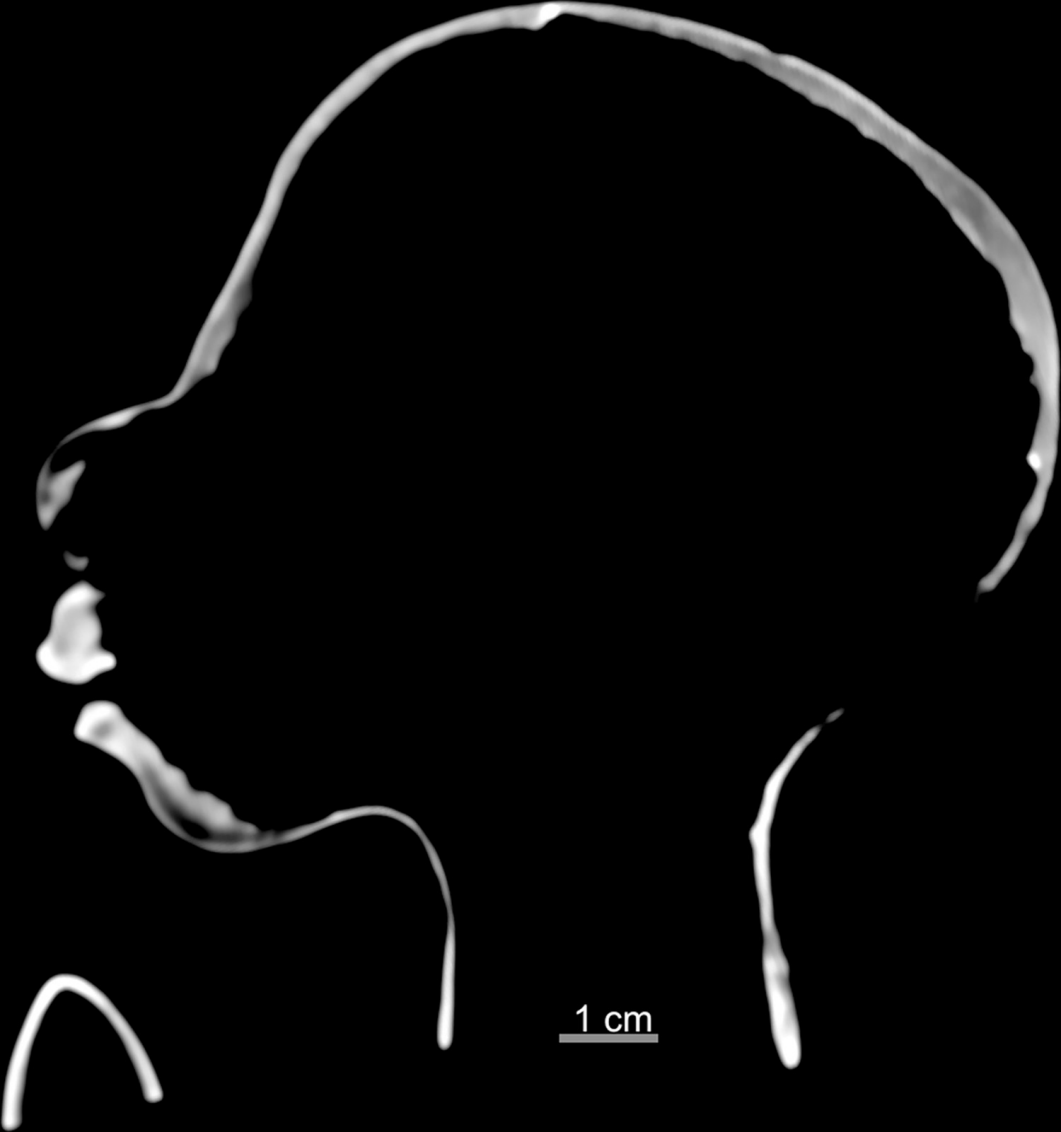
Comparative slice images of the scalp at different resolutions and magnifications. Mid-sagittal slice through the *tsantsa*–clinical CT scan. The scan has been windowed and leveled to maximize the visibility of the double hiding on the posterior part of the scalp.

**Fig 9:**
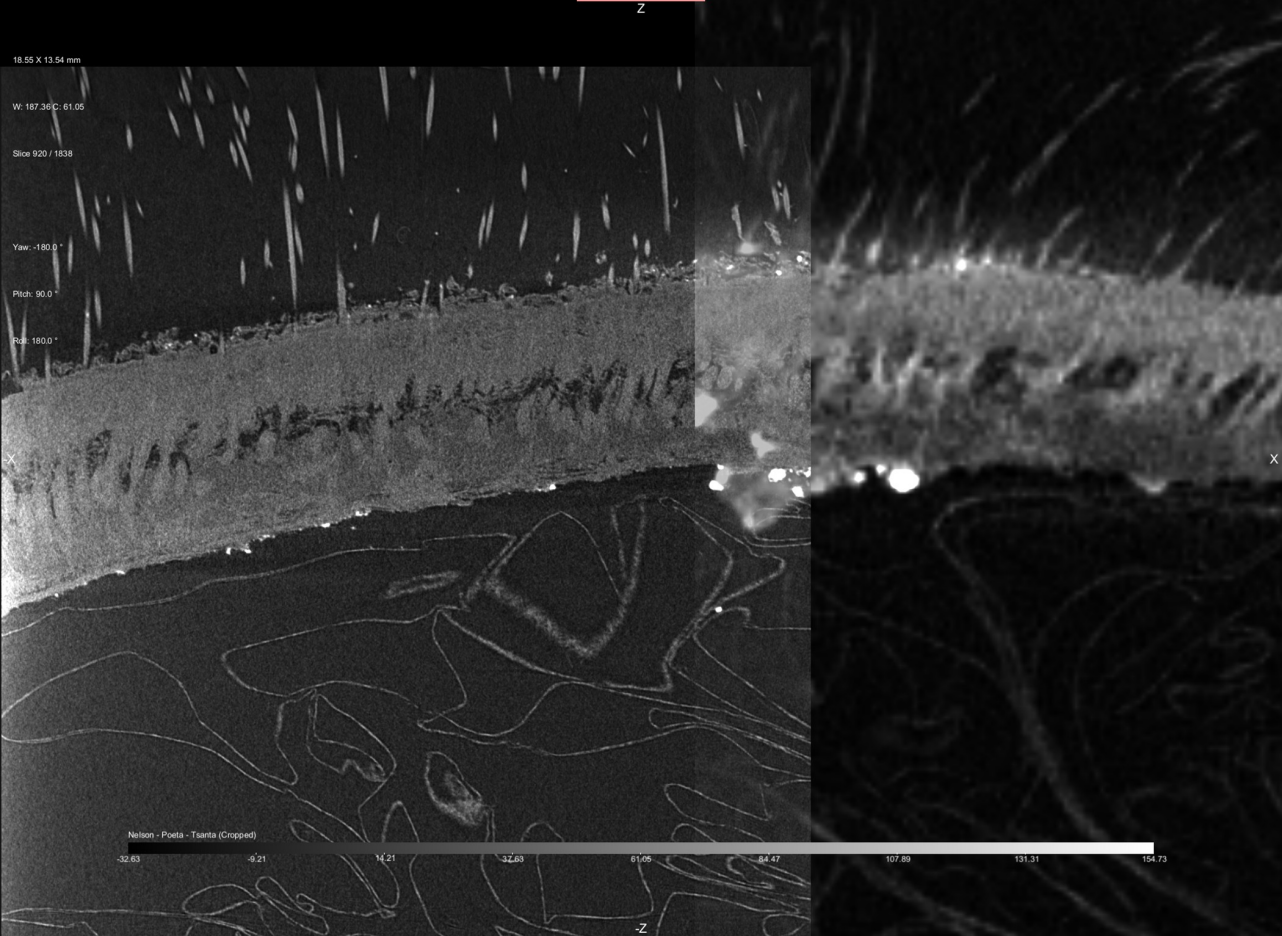
Slice of the co-registered micro-CT volumes at 14 μm (left) and 75 μm scan (right).

All three scans were used to assess the authenticity of the Chatham *tsantsa* based on the scalp (Fig 8). The clinical CT resolution provided little detail in the visualization of the dermal layers, with only a slight colour difference indicating separation of the layers, referred to as double hiding [14]. However, this is not the case for the two visualizations using micro-CT resolutions. The Chatham *tsantsa* clearly demonstrates double hiding as an air pocket is demonstrated on the 75 μm resolution scan (Fig 9), which was not clearly identifiable in the clinical CT scan. However, the details of the dermis and hair were still unclear. The 14 μm resolution scan proved to be the best for assessing the Chatham *tsantsa’s* scalp for authenticity (Fig 9). At this resolution, cross-sectional slices enable the visualization of individual shafts of hair and hair follicles. The hair follicles are embedded in the remnants of the dermis, some of which was apparently gelatinized in the preparation process, and the hair shafts can be seen to pierce the upper layer of skin, precisely how human hair follicles are embedded in the dermis and the shafts pierce through the epidermis. This higher resolution scan enables the accurate assessment of the *tsantsa’s* scalp, enabling the identification of natural hair growth. This scan also clearly demonstrates that the hair is real and not present in the form of human hair plugs.

**Fig 10:**
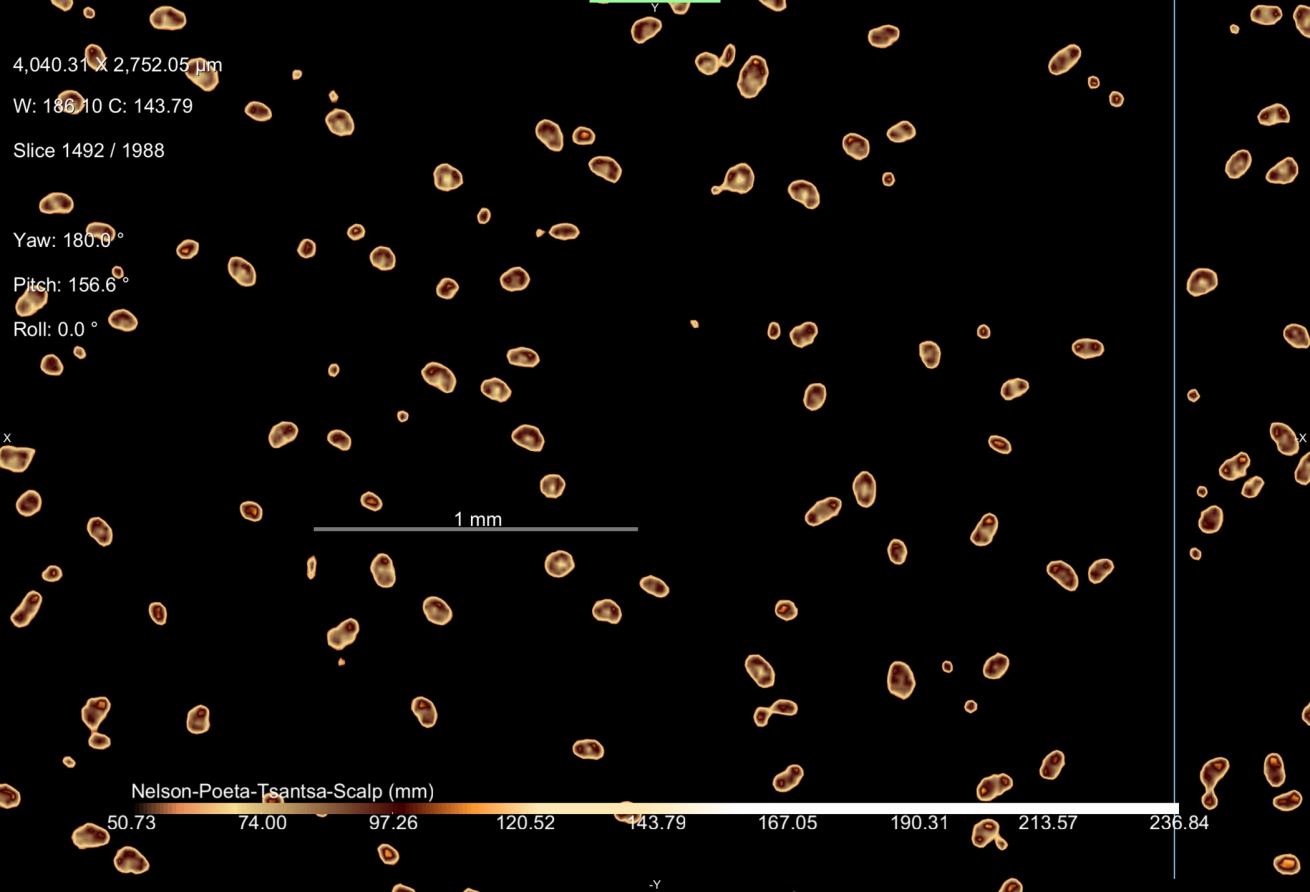
High resolution micro-CT slice through the hair shafts of the *tsantsa*.

It is possible that the head could be real hair, but derived from a sloth, as many tsantsas were made from sloths’ heads. However, sloth hair has some unique morphological properties to enable their symbiotic relationship with algae [37]. *Bradypus variegatus* (the brown throated 3-toed sloth) and *Choloepus didactylus* (the Hoffman’s 2-toed sloth), two sloth species native to the region occupied by the Shuar and Achuar, are differentiated from other mammals by lacking a medulla at the center of the hair shaft. *C*. *didactylus* is further identified by the existence of flutes or ribs running longitudinally down the shaft [37]. An examination of the cross-section of a hair shaft on the high-resolution micro-CT scan demonstrates a circular shape and the presence of a medulla (Fig 10), suggesting that this is human hair rather than that of a sloth and a 3D image of the hair demonstrates the scaled structure typical of human hair and not the fluted form of *C*. *didactylus* hair [37]. Note that the shafts are roughly circular or oval in cross-section, they do not have the flutes on the external surface that characterize sloth hair and they have a medulla. This image has had a false color look up table applied to enhance the visibility of subtle density differences in the image. Visual examination with light microscopy confirms the lack of fluting.The differentiation of human hair from other species of animals would be very difficult on the basis of the micro-CT. Other techniques from wildlife management [38] or forensics [39] would be required for that application.

## Discussion

The objective of this study was to examine a *tsantsa* using three different resolutions of clinical- and micro-CT scans in a correlative manner to assess whether the *tsantsa* was made from an actual human head, and if so, to examine key features that could determine whether it had been made for ceremonial purposes following authentic Shuar and Achuar beliefs and ceremonial practices, or if it had been manufactured for the tourist or museum commercial market. We presented a consensus description based on (often conflicting) original accounts of ceremonial preparation and highlighted four traits that have been used in recent studies that emerge from the consensus that should differentiate ceremonial from commercial *tsantsas*. The four traits were the stitching, the anatomy of the ear, the anatomy of the eye, and the anatomy of the scalp.

A clear, uninterrupted view of the incision cannot be assessed through a visual examination of a *tsantsa* as the hair is thick and long. While clinical CT resolution enables the judgement of incision shape by virtual removal of hair that covers the incision, only micro-CT resolution enables the assessment of the stitching. The Chatham *tsantsa’s* posterior incision exhibits an uneven course, which is consistent with a cut by a bamboo or lithic blade, but very precise stitchwork which appears to align with commercial work and materials [7]. The stitchwork is not visualized with the clinical CT resolution because the thread is thinner (ca. 200 μm) than the voxel size (293*293*500 μm).

The sewing of facial openings was a critical part of the ritual to protect the living from the soul escaping and is therefore an important trait for authentication of ceremonial origin. The eyelids may have remained pointing inwards after scraping, but the only stitching is very weak and commercial in appearance. Again, the stitching cannot be resolved on the clinical CT scans, but the micro-CT scan suggests that the eyes were stitched shut with modern thread in a non-traditional or “non-authentic” manner.

The clinical CT scan cannot resolve the details necessary to determine if the auditory canal exists, therefore, to determine if the *tsantsa* has true human ear anatomy or if it was constructed from an animal skin pierced and reshaped to mimic a canal. The lower resolution of the clinical scan blends the different tissue and inclusion densities instead of isolating and contrasting them as they appear in the micro-CT visualization. Clinical CT resolution is not reliable in identifying human ear anatomy; thus, it should not be used to determine the authenticity or the likelihood of a *tsantsa* being made from human remains unless combined with a DNA analysis, such as that completed by Baquero-Méndez et al. [4].

Clinical CT scan of ceremonial *tsantsas* demonstrate the presence of ‘double hiding’ in the scalp [18]. Double hiding is thought to result from the manufacturing processes that cause the reticular layer of the dermis (skin) to gelatinize and disappear, but the epidermis and hypodermis remain [11]. This taphonomic process is believed to create a pocket of air between the epidermis and hypodermis. Note that the clinical CT scan of this *tsantsa* presents inconclusive evidence for double hiding, while the high-resolution scan demonstrates that the degradation of the dermal layer was present, but incomplete, and it still enclosed the hair follicles. The long hair of the individual was said to be one of the most important features for a *tsantsa* as the hair is grown throughout an individual’s life without being cut and is said to contain the soul of the individual [1, 20, 24]. Men traditionally wore their hair longer than the women, so *tsantsa* hair that is over half a meter long is not uncommon [1].

Natural hair on animal skins used to create commercial *tsantsas* is sparse across the scalp and relatively short, a few inches long at most, whereas the Chatham tsantsa has hair that is 42cm long and densely clustered in the scalp region as would be expected if a human head was shrunken. This higher resolution micro-CT scan demonstrates the hair present on the Chatham *tsantsa* as natural human hair distributed throughout the scalp and not hair plugs such as those found on some commercial *tsantsas* that include hair at similar lengths [10]. The hair length, anatomical structure, and structural differentiation from sloth hair demonstrates that this *tsantsa* was created from a real human head.

### Benefits of combining multiple resolutions (correlative tomography)

Correlative *microscopy* is the registration of images from 2 or more 2D imaging modalities or resolutions into a single image. The objective is to take advantage of the properties of different imaging modalities, such as colour (e.g. light microscopy), density (e.g. backscattered SEM), and/or resolutions to highlight specific features that cannot be appreciated on any single image [40]. The most common form of correlative microscopy is Correlative Light and Electron Microscopy (CLEM) [41]. Correlative *tomography* is the registration of 2D images (e.g. histology) onto 3D volumes created by tomography (CT, micro-CT, MRI) [31] or the coregistration of 2 or more 3D volumes captured at different resolutions in order to highlight the hierarchical structure of biological structures [e.g. 42, 43] or metals [e.g. 44]. The term “multimodal imaging” has also been used to refer to these correlative approaches [44]. The combination of images taken at different resolutions is not new, as it has long been used for “scout and zoom” strategies for finding regions of interest at low resolution for interrogation at higher resolution with another imaging modality [31, 45]. However, the extension of the concept into the 3rd dimension and the increasingly sophisticated image processing and registration techniques that have recently become available, have led to a great expansion of applicability of the technique in the last decade.

In the case of this *tsantsa*, lower resolution scans were necessary to view the head as a whole, but the successively higher resolution scans were critical to see finer and finer details. Unfortunately, it is simply not possible to scan the entire head at the highest resolution. This is due first to the physical limitations of the geometry of the scanner, but also to the fact that the resulting dataset would be approximately 115 GB in size, which is currently well beyond the capacity of most computers (although this is rapidly changing). Thus, the most effective strategy would be to scan the whole head at the maximum resolution available (“scout”) and then to select individual areas of interest for higher resolution inspection (“zoom”).

## Conclusion

The Chatham *tsantsa* has been confirmed to be real human remains using multi-resolution computed tomography scans, but is likely not ceremonial, according to the consensus narrative of how ceremonial *tsantsas* were prepared. The assessment of key traits for authentication, including stitching, the anatomical structure and preparation of the eyes, the anatomical structure of the ear, and the minute anatomical structure of the scalp require advanced resolution from a micro-CT scanner. However, it is still not entirely clear if this is an authentic or a commercially produced *tsantsa*. The fact that it is a human head, the rough cut at the back of the skull, and double hiding are consistent with authentic ceremonial preparation. However, the use of modern thread in the stitching of the posterior incision, eyes, and lips suggest that it is a commercial production.

In fact, it may be that the division between ceremonial and commercial manufacture is more difficult to define than generally believed, as the practice of creating *tsantsas* likely exists on a spectrum rather than an either/or dichotomy. It is also likely that there were subtle variations between Indigenous communities’ practices throughout the Shuar and Achuar Peoples, as is reported in a current project that includes community representatives in Ecuador [4]. Finally, it is quite likely that commercial *tsantsa* makers were very good at their craft, closely mimicking the ceremonial *tsantsas*, or that Shuar or Achuar people themselves may have been creating the commercial *tsantsas*. However, ceremonial *tsantsas* should be more homogenous in appearance compared to commercial *tsantsas* as they were produced through traditional practices that were taught through generations of Shuar and Achuar people [4]. Further work is required to examine *tsantsas* that retain their original provenience at micro-CT resolution to explore these questions.

Thus, with micro-CT resolution, it is possible to identify whether a *tsantsa* has been created from real human remains. This is relevant to the question of using radiographic techniques to inform the curation and display of human remains, having the correct insurance on museum collections, and in repatriation efforts of the material culture and biological remains of Indigenous Peoples that are currently held in museum collections. CT and micro-CT technology’s non-destructive nature is a particularly desirable feature for the curatorial and conservation process. While more work is required to continue differentiating the ceremonial and commercial manufacture procedures of *tsantsas*, the inclusion of micro-CT imaging can aid one crucial aspect of ethical museum curation and display, whether a *tsantsa* was created from human remains.

However, clinical CT scans still offer the standard resolution for reconstructing a basic digital visualization of a *tsantsa* for a non-physical examination to lessen the risk of damage from repeated physical handling and physical examinations of each *tsantsa*. For example, clinical CT resolution provides enough detail to assess if the sutures are cut roughly or cleanly, if any material is found inside the head, and overarching anatomical features of a *tsantsa*. Also, if working with the Shuar and Achuar to construct culturally-appropriate and community-led exhibitions featuring the *tsantsas*, the clinical CT scans would be appropriate for the majority of the digital reconstruction wanted, unless also desiring the authentication process to be discussed as part of the exhibit. Using a clinical CT digital reconstruction also saves on the computer power needed to support such a technical exhibit. Overall, clinical resolution is an appropriate starting point for any investigation into the origin and manufacture of *tsantsas*, but the micro-CT reconstructions are required for examining the material and assessing specific features.

## Supporting information

S1 Table Features used to differentiate ceremonial and commercial *tsantsas*. (DOCX)
