# Supplementary material for: Correlative tomography and authentication features of a shrunken head (tsantsa)
Source: PLoS One. 2022 Aug 3;17(8):e0270305. doi: 10.1371/journal.pone.0270305 (PMC9348654; doi:10.1371/journal.pone.0270305)
Supplement: S1 Table — (DOCX) [file pone.0270305.s001.docx]

**Supporting Information**

| Ceremonial *Tsantsa* | Commercial *Tsantsa* |
| --- | --- |
| 1. Size approximately equal to a clenched human fist | 1. Variable scale |
| 2. Posterior median sutured incision | 2. Variable incision locations |
| 3. Sutures are often wide and uneven – performed using a thick and inflexible flat bamboo needle with coarse chambira vegetable fibre string | 3. Stitches are usually more precise, discreet and consistent – due to access to finer, sharper, metal needles and thinner suture threads |
| 4. Loop of flexible vine is sewn into the neck – if absent, traces of suturing are often evident | 4. No supporting vine at the neck structure |
| 5. Eyelids are tightly drawn into the head and sutured shut | 5. Can be variable, but the eyelids are often sutured with the upper lid positioned over the lower lid |
| 6. Three mouth perforations from chonta pin application – sometimes retained and lashed together with chambira | 6. No, or a variable number of perforations mark the mouth. If pins are present, they are not always chonta wood |
| 7. The mouth pins are classically replaced with intricately woven string tassels applied to the mouth at a length equal to the scalp hair. Several horizontal red bands of achiote are painted, but these can fade over time | 7. Tassels are often not attached. If present, atypical colourants, materials and knots for securing them, may be present |
| 8. Skin browned from carbon staining | 8. Skin remains unstained, presenting as a grey or yellow colour |
| 9. Facial down removed | 9. Facial down is sometimes maintained |
| 10. Skin is polished | 10. Skin can sometimes present a dull, rough texture |
| 11. Narrow heads, often presenting a ‘pinched’ impression at the temples | 11. Care was often taken to retain more natural proportions. The curio trade was preoccupied with human heads, thus care was taken to preserve its human appearance |
| 12. Common distortions include the forced extension of the mouth, and the spreading and upturning of the nose | 12. Forced distortions were typically avoided |
| 13. Typically long scalp hair is present, with no facial hair | 13. Scalp hair can vary in length. Facial hair is often maintained |
| 14. One or two perforations mark the crown, with one fitting a vegetable fibre string suspension cord (woven into a five-loop braid) that is secured within the head by a small wooden pin. The cords are long enough for adornment about a person’s neck | 14. Heads are not always perforated to fit a cord. If cords are fitted, they can be produced from a variety of different materials, woven differently, overly decorated and of an inappropriate length for personal adornment around someone’s neck |
| 15. Piercings that would typically present at the earlobes of SAAWC victims were not always decorated. Toucan feather ear danglers and/or wooden tubes/pins are however common | 15. Headbands, necklaces and any ornamentation consisting of beads, seeds, or portions of seeds, are not documented in SAAWC material culture |
| 16. Skins tend to be dense and of considerable weight | 16. Skins can sometimes be thin, fragile and lightweight |
| 17. Triangular-shaped nostrils | 17. Circular-shaped nostrils |
| 18. Double hiding present 100% | 18. Double hiding rarely present |
| 19. Rounded cranium in sagittal cross-section view | 19. Mis-shaped cranium in sagittal cross-section view |

**S1 Table. Features used to differentiate ceremonial and commercial *tsantsas*.**

Based on the table found in Houlton TM, Wilkinson CM. Recently identified features that help to distinguish ceremonial tsantsa from commercial shrunken heads. Journal of Cultural Heritage. 2016 Jul 1;20:660-70.
